# Diagnosis on Transport Risk Based on a Combined Assessment of Road Accidents and Watershed Vulnerability to Spills of Hazardous Substances

**DOI:** 10.3390/ijerph15092011

**Published:** 2018-09-14

**Authors:** Emerson Ribeiro Machado, Renato Farias do Valle Junior, Teresa Cristina Tarlé Pissarra, Hygor Evangelista Siqueira, Luís Filipe Sanches Fernandes, Fernando António Leal Pacheco

**Affiliations:** 1 Laboratório de Geoprocessamento, Instituto Federal do Triângulo Mineiro, Campus Uberaba, Uberaba MG 38064-790, Brasil; emersonrimac@oi.com.br (E.R.M.); renato@iftm.edu.br (R.F.d.V.J.); 2 Departamento de Engenharia Rural, Faculdade de Ciências Agrárias e Veterinárias, Universidade Estadual Paulista, Jaboticabal SP 14884-900, Brasil; teresap1204@gmail.com (T.C.T.P.); hygorsiqueira@yahoo.com.br (H.E.S.); 3 Centro de Investigação e Tecnologias Agroambientais e Biológicas, Universidade de Trás-os-Montes e Alto Douro, Ap 1013, 5001-801 Vila Real, Portugal; lfilipe@utad.pt; 4 Centro de Química de Vila Real, Universidade de Trás-os-Montes e Alto Douro, Ap 1013, 5001-801 Vila Real, Portugal

**Keywords:** environmental vulnerability, multi-criteria spatial analysis, risk management tool, hazardous substance, road accidents

## Abstract

Roads play an important role in the economic development of cities and regions, but the transport of cargo along highways may represent a serious environmental problem because a large portion of transported goods is composed of dangerous products. In this context, the development and validation of risk management tools becomes extremely important to support the decision-making of people and agencies responsible for the management of road enterprises. In the present study, a method for determination of environmental vulnerability to road spills of hazardous substances is coupled with accident occurrence data in a highway, with the purpose to achieve a diagnosis on soil and water contamination risk and propose prevention measures and emergency alerts. The data on accident occurrences involving hazardous and potentially harmful products refer to the highway BR 050, namely the segment between the Brazilian municipalities of Uberaba and Uberlândia. The results show that many accidents occurred where vulnerability is high, especially in the southern sector of the segment, justifying the implementation of prevention and alert systems. The coupling of vulnerability and road accident data in a geographic information system proved efficient in the preparation of quick risk management maps, which are essential for alert systems and immediate environmental protection. Overall, the present study contributes with an example on how the management of risk can be conducted in practice when the transport of dangerous substances along roads is the focus problem.

## 1. Introduction

According to data from the National Confederation of Transport (http://www.cnt.org.br/), in Brazil about 61% of cargo and 95% of passengers are transported via highways, indicating that this transportation sector is economically relevant at the national scale, handling a substantial amount of financial resources [1]. Despite the recognized importance of highways for the country’s economic development, road transport involves a diversity of cargo types, including dangerous products, which renders this activity a preoccupying risk of environmental and social impacts. The risk associated to the transport of a dangerous product depends not only on the substance being transported but also on the road network characteristics, weather conditions, driver skills and population concentration along the selected routes [2]. The potential impacts of accidents related to road transport of hazardous products were discussed in various studies, which triggered social concerns and were helpful as guides to the conception and implementation of adequate corrective and preventive actions [3]. However, before implementation of any specific measure, a correct diagnosis on vulnerability and risk is required.

The Multi Criteria Analysis (MCA) method embedded in a geographic information system (GIS), also known as Spatial MCA, is a computational tool that can assist the preparation of vulnerability and risk maps for decision making on the planning and operation road transport networks [4,5,6], as well as in many other environmental applications [7,8,9,10,11,12]. In the study of [5] MCA was used to determine the vulnerability of soil and water resources in rural basins, while in the study of [6] it was used to model the environmental risk of accidents involving the transport of dangerous products based on static and dynamic data. The work group of Van [13] developed a method to evaluate statistical data on road accidents involving dangerous products, from which it was possible to generate a global risk map. Regardless the method on which the vulnerability, the risk or the hazards are assessed, from a safety standpoint road risk management requires the implementation of methods that are capable to provide a quick diagnosis on the potential environmental impact of an accident involving the spill and leaching of dangerous substances from a road. The challenge is therefore to develop and validate robust but expeditious diagnostic tools. The resort to statistical data on road accidents demands a significant time span dedicated to monitoring, while the generation of vulnerability maps cannot stand alone as method to identify the risky areas [2]. According to [14], besides the potential lack of reliable numbers the management of road transport risk based on statistical data requires the capability to evaluate, in a short period of time, the diversity of transported materials, and the possible environmental consequences related to their road spill. This evaluation becomes even more complex given the multiplicity of road accident circumstances. The route to follow is therefore to combine hazard assessments (road accident counts) with vulnerability assessments at site and catchment scales.

The main purpose of this study is to combine a method already used to determine the environmental vulnerability of areas adjacent roads to spills of dangerous products [15] with road accident data, in a manner that becomes possible to analyze the accident scenario immediately after the occurrence and thus to implement an alert system whereby corrective measures can readily be triggered, such as the sending of resources, isolation of a certain area, withdrawal of the population, protection of springs, soil or water decontamination, among others.

## 2. Area of Study

The area where the risk management method is to be implemented is located between the municipalities of Uberaba and Uberlândia, in the Brazilian State of Minas Gerais, mesoregion of Triângulo Mineiro. The municipality of Uberlândia is the second most populous in Minas Gerais, and the 30th in Brazil, with 676,613 inhabitants according to the Brazilian Institute of Geography and Statistics—IBGE [16]. The municipality of Uberaba has a population of 328,272 inhabitants, as estimated in 2017, being the 8th largest municipality in the state and the 81st in the country [17]. The sector under study comprises a 97-km segment of highway BR 050, from km 77 at the junction of BR 050 to kilometer 174 at the intersection with BR 262. The BR 050 is an important connecting corridor between the central-west and southeast regions of Brazil, receiving the flow of several highways that cross the country from north to south, namely the traffic from the Federal Highways BR 365/452/455/497. The BR 050 highway was built in the 1970s with the main purpose to connect the capital of the country to the Port of Santos SP. In 2010 the segment under study was doubled, increasing the highway capacity.

**Figure 1:**
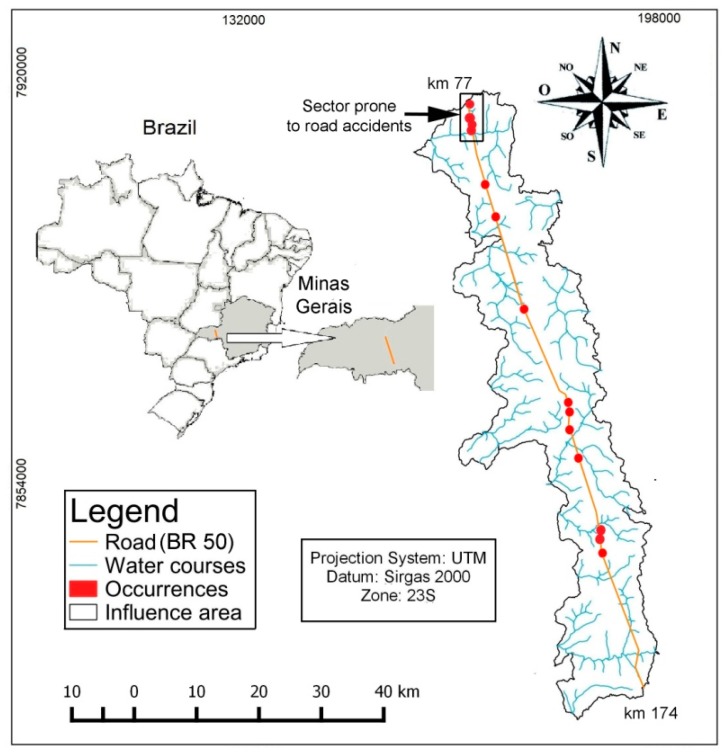
Geographic location of the studied BR 050 highway segment, in Brazil and Minas Gerais State, with representation of intercepted water courses and distribution of road accidents involving spills of hazardous substances. In the northern and central parts of this segment the road was built nearly along a water divide. In these sectors the water channels are likely to be equally vulnerable to contamination at both sides of the road, because the spill of a harmful substance will potentially leach in both directions. For similar reasons, in the southern part the water channels from the west side are potentially more vulnerable than the channels from the east side.

Along the studied segment the BR 050 highway intersects several watercourses that drain small watersheds (Figure 1). The climate of Triângulo Mineiro region is qualified as Aw according to the Köeppen classification. The Aw climate is a tropical mega thermal climate, with winter droughts and an average temperature for the coldest month around 18 °C. Precipitation is characterized by annual averages of 1200 to 1450 mm, according to the climatic norm of Brazil (1961–1990) published by the National Institute of Meteorology [18]. The dry period runs from May to September and the wet period from October on [19]. As regards geomorphology, the Triângulo Mineiro is located in the so-called “Plateaus and Mesas of the Paraná Sedimentary Basin”, which comprise the “Northern Plateau sub-unit” [20]. Geologically, this plateau is characterized by deposits of Uberaba, Marília and Vale do Rio do Peixe formations belonging to the Bauru group; Serra Geral formation belonging to the São Bento group; and undifferentiated dendritic and/or lateritic [21].

## 3. Materials and Methods

**Figure 2:**
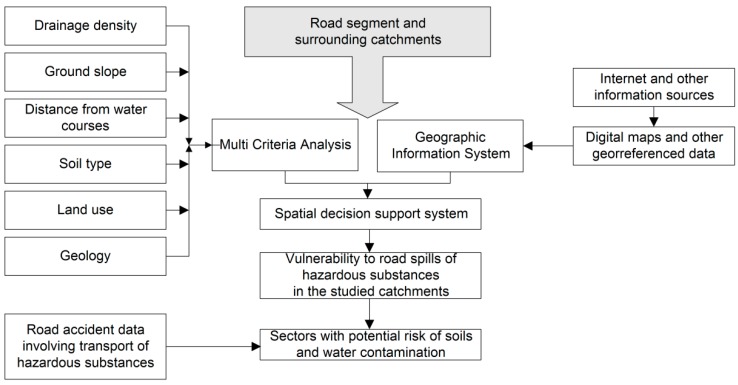
Flowchart illustrating how contamination risk has been assessed in the present study, associated with spills of hazardous substances following a road accident.

The method to investigate road accident scenarios based on the assessment of environmental vulnerability and road accident data related to transport of hazardous substances is illustrated in Figure 2 in the form of a workflow. The Multi Criteria Analysis represented in this diagram and used to assess environmental vulnerability has been developed and presented in the previous work of [15]. The coupling of those results with road accident data is performed in this study. The method developed in the earlier work of Machado and co-authors is briefly described in Section 3.1. The complement related to analysis of road accident data is described in detail in Section 3.2 and Section 3.3. Both methods were implemented in Geographic Information System (GIS), frequently used in environmental studies [22,23,24,25,26,27,28,29,30,31,32,33,34]. In the present study, the specific GIS was IDRISI Selva software [35] that resorted to various sources of digital information, mostly public institution websites (e.g., http://www.webmapit.com.br/inpe/topodata/ for topographic data or https://earthexplorer.usgs.gov/ for land use/land occupation data). The full inventory of information sources is listed in [15].

### 3.1. Determination of Vulnerability

**Figure 3:**
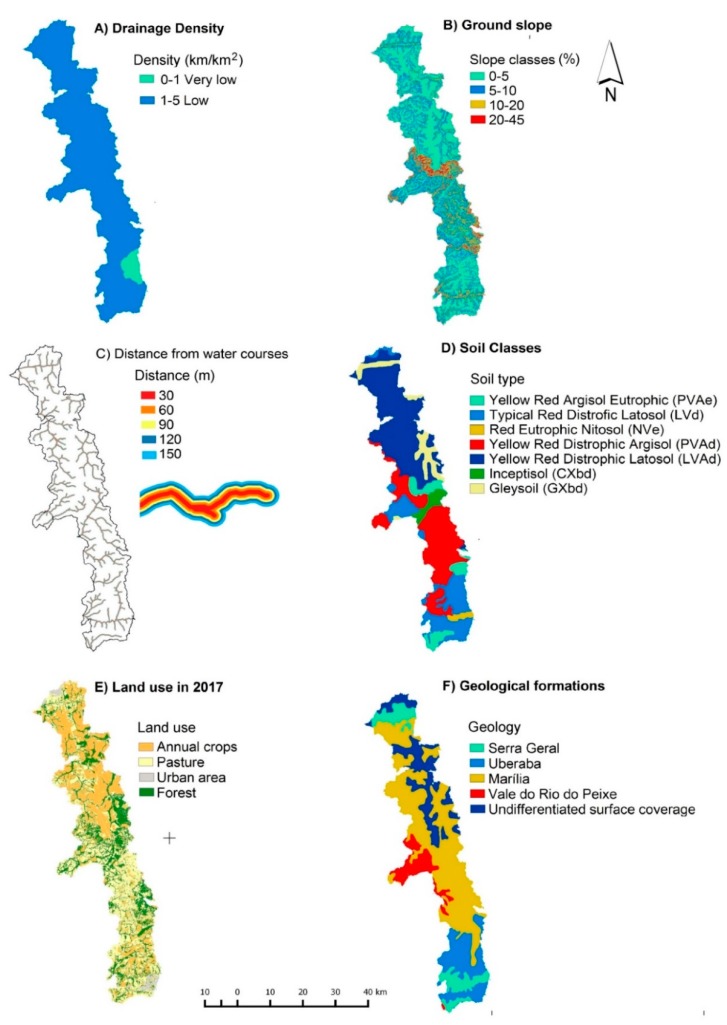
Spatial distribution of vulnerability-relevant factors included in the Multi Criteria Analysis. Adapted from [15].

As mentioned, an environmental vulnerability evaluation along the BR 050 highway was accomplished by the authors of this study in a previous publication [15]. To complete the task, a Multi Criteria Analysis (MCA) embedded in a geographic information system was applied to the areas under direct influence (within a 210 m buffer from each margin of the highway) and indirect influence (within the limits of the micro basins along the 97 km segment) of spills of dangerous products. The MCA approach is a four-step process, which involves (1) selection of factors to describe vulnerability, with subsequent normalization of factor scales into a common dimensionless range, and elaboration of raster maps that describe the spatial distribution of normalized factors; (2) the allocation of a weight to each factor; (3) the weighted combination of factors to compose a final vulnerability map; and (4) a sensitivity analysis of vulnerability results based on scenarios [36]. The four steps are briefly outlined in the next paragraphs:

*Step 1*: In the study of [15] the vulnerability maps were based on the following factors: (1) drainage density, (2) distance from water courses, (3) ground slope, (4) soil type, (5) land use/occupation, and (6) geology (Figure 3; Table 1, part (a)). These factors were selected because they are comparable to key variables of drainage models and flow routing algorithms that describe the detachment and transportation of pollutants in catchments [5]. The normalization of factor classes was based on a byte-level interval (0 to 255), which linked a 0 level to the least important class and 255 level to the most important class (Table 1, part (b)). The association of factor classes to levels of importance (ratings) was based on the authors’ personal experiences about vulnerability assessments.

*Step 2*: The allocation of weights was based on the Analytical Hierarchy Process (AHP; [37]) whereby the user (or a group of experts) assign a relative importance to each factor based on pairwise comparisons with the other factors, and then this hierarchy is processed in the AHP algorithm to obtain a set of optimized levels of importance (weights). Because the attribution of weights can be subjective, in the study of [15] a sensitivity analysis was performed (step 4 below) where various factors were given the largest relative importance and hence maximum weights.

**Figure 4:**
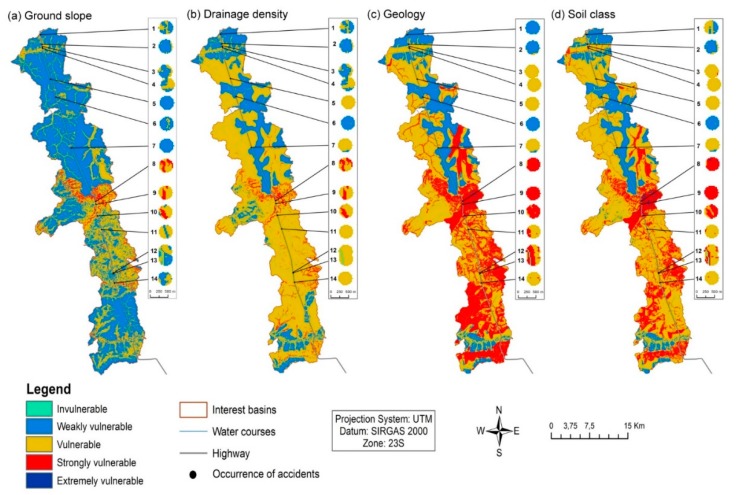
Vulnerability maps of the intercepted water course catchments, highlighting the vulnerability at the road accident sites (also termed hazard; labeled circles). The maps are outcomes of a Multi Criteria Analysis where vulnerability-relevant factors ground slope (map (**a**)), drainage density (**b**), geology (**c**) and soil type (**d**) were given the largest weight [15]. The concomitant effects on vulnerability are reflexes of factor heterogeneity across the studied area. The largest effect occurs when factors geology or soil type are maximized highlighting the importance of these factors.

*Step 3*: The overall vulnerability was calculated by Equation (1), implemented in the GIS software using map algebra tools and factor maps in raster format (Figure 4):

(1) $$S_{i}=\overset{p}{\underset{i=1}{\sum }}w_{j}X_{ij}$$

where *S_j_* represents the vulnerability at pixel *i*, *w_j_* represents the weight of the factor *j*, and *X_ij_* represents the normalized value of factor *j* at pixel *i*. The *S_i_* values are reclassified into five classes using the same byte-level range: Invulnerable (0–50), Weakly vulnerable (50–100), Vulnerable (100–150), Strongly Vulnerable (150–200), and Extremely Vulnerable (200–250).

*Step 4*: The sensitivity of *S* to changing factor weights was evaluated through generation of four scenarios where one of these factors has been given the largest relative importance maximizing its weight. The factors that have been given maximum weights were drainage density, ground slope, soil type and geology. The scenarios were created because the aforementioned factors are heterogeneous across the studied region and in that context associated with an ample range of scores. For these reasons there is no easy way to define a universal hierarchy to describe the importance of each factor. For example, the studied segment of BR 050 highway is contrasting as regards ground slope, because the north and south sectors are occupied by plains linked to low vulnerability while the central part is mountainous and linked to high vulnerability. In a scenario that maximizes the importance of ground slope, these contrasting topographic features will be highlighted in the final vulnerability map, while being smoothed otherwise. The same rationale holds for the other factors as well. Vulnerability in the four scenarios is illustrated in Figure 4a–d.

As expected, when factor ground slope is maximized (Figure 4a) the vulnerability map shows a central area with high vulnerability bordered to the north and south by areas with low vulnerability. However, it is evident from analysis of Figure 4c,d that rising the role of geology or soils in the vulnerability assessment results in larger overall vulnerability. It is also worth to note that the BR 050 highway in the studied segment was built nearly along a water divide in the northern and central sectors but away from it in the southern part (Figure 1). For that reason, spills of dangerous substances in the northern-central parts of the segment will potentially affect the water courses in both sides of the highway while in the southern part spill drainage will primarily affect the western channels.

### 3.2. Occurrence Data Involving Hazardous and Potentially Harmful Products to the Environment

The data on accidents involving hazardous and potentially harmful products in the studied segment was obtained from the Concessionaire who manages approximately 700 km of BR 050 highway. The collected data is summarized in Table 2. It is important to note that some products represented in the table are not classified as dangerous by the United Nations (http://www.unece.org/trans/danger). These products are identified as “not applicable” under the heading “UN Code” (Column 3). However, because the road spill of these products can contribute significantly to soil and water contamination in accident scenarios, they were used in the present study of risk management. The accident data was compiled from operational resources such as Operational Control Center (OCC), Traffic Inspection and Mechanical Rescue Vehicles, Emergency Medical Service Vehicles (rescue and salvage), Closed Circuit TV and Radio Communication System. Through the OCC, all the occurrence data are recorded using the software Kria Operational Control for Highways. Among other issues, the recording involves the generation of a GIS database through the conversion of site details (i.e., the exact kilometer of the occurrence) into geographical coordinates (last columns of Table 2). Besides generation of data the software releases management reports according to the periodicity and type of occurrence desired, allowing analysis, treatment and decision making. The data for the present study spans the period from July 2014 to December 2017, which represents a 2 years and 6 months interval. Figure 1 shows the distribution of occurrences (red circles) involving hazardous products in the studied segment, obtained through the Concessionaire. We recognize that the data record is not long to provide a clear image of the situation, but are confident that enables a preliminary view.

### 3.3. Environmental Vulnerability at Occurrence Sites (Risk)

The vulnerability around the road accident sites listed in Table 2 was assessed by the IDRISI Selva software [35], taking into account the four predefined scenarios (Figure 4). The IDRISI Selva software embeds a set of tools capable to determine the environmental vulnerability of an area according to the necessary steps. The vulnerability profile of each site was defined through the following steps: (1) a 200 m buffer was drawn around the site. This area covers the environmental resources immediately affected after the occurrence of spills; (2) The buffers were plotted over the four vulnerability maps (Figure 4a–d); (3) For each vulnerability scenario, the area related to a vulnerability level (e.g., strongly vulnerable) was determined using raster map operations.

Steps 1–3 were repeated for all the vulnerability levels and all sites, and aggregated per vulnerability level in each scenario. To distinguish the vulnerability evaluated within the studied segment (catchment scale) from the vulnerability evaluated around the road accident sites (buffer scale) the latter was termed hazard. Having determined the hazard area within the 14 buffers, the risk of soil and water contamination is estimated for every vulnerability level using the formula:

(2) $$R_{j}=\frac{H_{j}}{V_{j}}=\frac{Ab_{j}}{A_{j}}$$

where *R_j_* is the risk for level *j*, *H_j_* is the hazard for level *j* evaluated within the 14 buffers and represented by the corresponding area (*Ab*_j_, in percentage of total buffer area), and *V_j_* is the vulnerability for level *j* evaluated within the studied segment and represented by its area (*A_j_*, in percentage of segment area). If the *R_j_* value is >1 then road accident sites are considered risky at that level. If sites are risky for the preoccupying levels (e.g., “strongly vulnerable” or “extremely vulnerable”) then the implementation of prevention and alert systems should be mandatory. These systems should also be considered for the “vulnerable level”. The analysis of risk can be refined, which means executed site by site. In this case, the *Ab_j_* represents the area of hazard level *j* within the specific site, in percentage of buffer area. It is worth mentioning that, besides hazard incidence and medium vulnerability the risk of soil and water contamination by dangerous substance, including public health issues, also depends on the extension of contaminant propagation, the amount and chemical properties (toxicity) of the spilled product, and the proximity of human presence [38]. Toxicity and proximity to urban centers will not be addressed in this study, because the vulnerability assessment on which the risk analysis is standing has been focused on the protection of environmental resources, soils and water.

## 4. Results and Discussion

### 4.1. Accident Count over the Monitored Period

**Figure 5:**
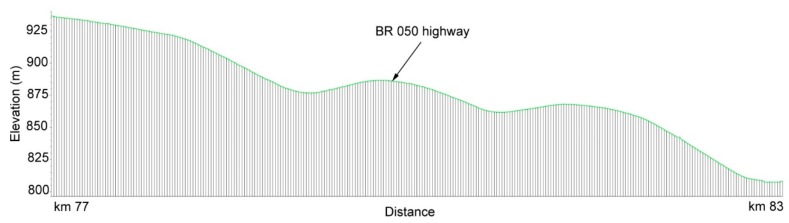
Elevation profile of the BR 050 highway segment involved in a large number of road accidents. This segment is located between km 77 and km 83 of the highway, as illustrated in Figure 1. The elevation profile was generated using the Google Earth software. The accidents are mostly caused by fast-speed traffic in a relatively steep-slope road.

A total of 14 accidents were reported to the monitoring system within the studied period (Table 2). Among these occurrences, 11 involved the transport of hazardous products and three the transport of products potentially harmful to the environment such as vegetable oil, limestone and cement. The largest number of episodes occurred in the morning (from 06:00 to 12:00, 35.71% of occurrences), being followed by the afternoon (from 12:00 to 18:00, 28.57%), night time (from 18:00 to 00:00, 21.43%), and early morning (from 00:00 to 06:00, 14.29%). As regards seasonality, there were nine occurrences in the rainy season (64.29%) and five in the period of low or no rainfall. The results also show that one quarter (28.57%) of all accidents occurred in the section between km 77 and 83 of BR 050. This section is characterized by steep slopes, but paradoxically is also distinguished by fast-speed traffic. Figure 5, generated in Google Earth, shows the elevation profile of km 77–km 83 section, with an elevation of 932 m at km 77 and 810 m at km 83 (2% slope, on average). Ferreira [39] studied the causes of road accidents with hazardous products in the State of São Paulo, Brazil, and observed a greater predominance of accidents in the afternoon (between 12:00 and 18:00) and that the routes between petrochemical poles also influence the number of accidents, due to the greater flow of vehicles transporting dangerous products along these routes. Overall, the results obtained in this study as well as by other authors [2] demonstrate the heterogeneity of factors influencing the incidence of accidents with hazardous products, which hampers the selection of priority sections for hazard management. The alternative path to follow relies on combining the assessment of hazard distribution with environmental vulnerability assessments, and hence moving from a conventional hazard management to the more integrated approach that is risk management. The assemblage of hazard and vulnerability data into a common framework of risk data, especially if using a geographic information system to accommodate and process the maps and associated attribute tables, has the additional virtue to help finding priority sections for management, because risky areas are fewer and smaller than the sum of hazard and vulnerable areas.

### 4.2. Vulnerability, Hazard and Risk

The 200 m-buffers around the 14 accidents sum a hazard area of approximately 168.27 hectares. The buffers were defined because large areas around the accident sites can be affected by the road spills, even extending to the entire watershed. Table 3 summarizes the results obtained for vulnerability, hazard and risk in the four predefined scenarios of factor maximization, respectively expressed as vulnerable areas within the BR 050 segment watersheds (*V*), hazard areas within the 200 m buffers surrounding the accident sites (*H*) and the percent ratio between *H* and *V* (*R* = *H*(%)/*V*(%)). The *V* areas were evaluated within the maps of Figure 4a–d, while the *H* areas were measured within the buffer areas (labeled circles) represented in the same figures. For example, for vulnerability level “strongly vulnerable” (red color in Figure 4) the *V* area is 4337.79 hectares (3.4% of road segment area; Table 3) in the ground slope scenario (Figure 4a), while raises to 40,073.87 hectares (31.4%) in the geology scenario (Figure 4c). The corresponding *H* areas are 11.50 hectares (6.83%) and 48.75 hectares (28.97%). According to Equation (2) this gives a risk *R* = 2.01 for the ground slope scenario and *R* = 0.92 in the geology scenario. In case ground slope is adopted as reference scenario for decision making on soil and water protection, then road accident sites located where the environment is strongly vulnerable are considered risky because *R* > 1. In general, as regards vulnerability the areas were mostly classified as weakly vulnerable or vulnerable, for the ground slope and drainage density factors, and as vulnerable or strongly vulnerable for the soil class and geology factors. The coverage by extremely vulnerable areas or invulnerable areas was insignificant. The results obtained for the areas where the accidents have occurred (hazards) follow the general results obtained for vulnerability, because the percentage of area ascribed to the vulnerability classes are similar in both cases. The exceptions occur for the scenarios where ground slope or drainage density factors were maximized, because in some cases the areas where the accidents have occurred are more vulnerable than the general vulnerability areas in those scenarios. As mentioned above, for the scenario that maximized ground slope the areas classified as strongly vulnerable along the highway watersheds represent *V* = 3.4% of the total watershed area while the homologous areas around the accident sites represent *H* = 6.83%. The same holds for the strongly vulnerable areas in the scenario that maximized the drainage density factor, which rise from *V* = 4.06% to *H* = 6.83%. Put another way, the strongly vulnerable areas in these two scenarios can be classified as risky, because *R* = *H*/*V* > 1 in both cases, namely 2 and 1.7 (Equation (1)). In that context, these vulnerability levels and corresponding areas of influence would deserve special attention in risk management plans.

Figure 4a–d display the vulnerability maps obtained at the 14 sites where the accidents occurred during the studied period. The maps also represent the surrounding watersheds, because they can also be environmentally affected. In all cases, these figures provide visual information for rapid environmental risk assessment of accident sites, enabling immediate prevention and alerts for the sites classified as vulnerable or strongly vulnerable. The representation of accident sites in a color scale related to environmental vulnerability validates the method of [15] as support for an expeditious risk management tool, and hence represents the achievement of a proposed objective. In that context, it is important to note the large number of accident sites located in strongly vulnerable areas, especially in the southern sector watersheds and when the focus of vulnerability is put on the catchments’ soil and geologic characteristics. An environmental alert is due in these cases to ensure the safety of soil and water quality within the involved watersheds.

## 5. Conclusions

The management of road transport risk involving hazardous substances is a challenging exercise because the factors influencing this variable depend on the vulnerability of the medium as well as on a myriad of road accident causes and circumstances. The challenge also results from the fact that, to be effective, a risk management tool needs to release the relevant information on local vulnerability immediately after the occurrence of an accident that spilled a dangerous product over the road. In this study, a combination of vulnerability and hazard assessments proved efficient to readily identify risky sections in a segment of highway BR 050 located in Brazil, which correspond to areas with a larger incidence of accidents located on strongly vulnerable areas. In these areas, the risk is mostly determined by ground slope and drainage density. The study could also depict accident sites and associated influence buffers as colored circles to readily represent vulnerability at site scale. It became evident at first sight that the southern sector of the highway BR 050 segment requires closer attention as regards environmental risk. In case of accident, soil and water contamination is highly probable because soils and geological formations are barely capable to sustain the propagation of contaminants in that sector. Overall, the study proved efficient in providing a comprehensive diagnosis on contamination risk along the studied road, as well as in providing clues about sectors of the highway requiring particular attention from risk managers.

## Figures and Tables

**Table 1:** (**a**) Factors used by [15] in the Multi Criteria Analysis of environmental vulnerability related to road accidents along the studied segment of BR 050 highway involving the transport of hazardous substances; (**b**) Normalization of factors within the Multi Criteria Analysis—MCA (step 2) designed to evaluate soil and water vulnerability along roads. The higher the value of a normalized factor the greater its importance for vulnerability. The MCA model was applied to a segment of BR 050 highway where transport of hazardous substances is intense and spills of those products following a road accident can cause severe damage to the surrounding environment. Adapted from [15].

**(a)**		
**Environmental Factors**	**Accident Scenario Implications**	
Ground slope	Factor that describes important aspects related to the control of erosion, transport of sediments and contaminants.	
Drainage density/distance from water courses	It describes factors related to the likelihood of water resources and biotic environment contamination.	
Geology	Factors related to likelihood of contamination, socioeconomic impact and the extent of damage in accident scenarios.	
Soil Classes/land use or occupation	It exposes factors related to the likelihood of soil and groundwater contamination and contaminant movement in accident scenarios.	
**(b)**		
**Factors**	**Values**	**Normalized Values**
Drainage density (km·km^−^²)	Very low	0–1
	Low	1–5
	Medium	5–13
	High	13–15
	Very high	>15
Distance of water course (m)	30	255
	60	175
	90	115
	120	75
	150	50
Ground slope (%)	0 a 5%	25
	5 a10%	75
	10 a 20%	125
	20 a 45%	255
Soil classes	Latosol	100
	Acrisol	150
	Nitisol	180
	Gleysol	200
	Cambisol	250
Land use and occupation	Annual crops	75
	Pasture	125
	Forest	200
	Urban Area	255
	Undifferentiated surface coverage	50
	Serra Geral	100
	Vale do Rio do Peixe	150
	Marília	200
	Uberaba	255

**Table 2:** Occurrences involving dangerous products in BR 050 during the monitored period. Symbols: UN—United Nations; UTM—Universal Transverse Mercator (coordinate system); X, Y—planimetric coordinates of the accident.

Date	Product	UN Code	Time	Kilometer	UTM—Zone 23 S	
					X	Y
09/29/14	Diesel oil	1202	13:04:00	082 + 180	161,681	7,898,073
10/09/14	Ethanol	1170	15:07:00	149 + 500	181,617	7,834,447
10/30/14	Diesel oil	1202	21:32:00	096 + 500	165,445	7,884,280
01/23/15	Toluene	1294	02:21:00	111 + 500	169,793	7,869,927
08/31/15	Ethanol	1170	15:52:00	152 + 120	181,988	7,831,857
09/12/15	GLP	1075	05:35:00	078 + 340	161,054	7,901,861
10/29/15	Oil S10	1202	11:30:00	081 + 800	161,619	7,898,448
10/14/16	Hydrated alcohol	1170	06:04:00	091 + 200	163,904	7,889,351
02/10/17	Diesel oil	1202	12:37:00	129 + 100	176,754	7,854,079
07/17/17	Hydrochloric acid	1789	06:31:00	149 + 300	181,607	7,834,653
10/24/17	Vegetable oil	not applicable	10:40:00	136 + 600	178,378	7,846,904
10/25/17	Limestone	not applicable	08:45:00	132 + 540	177,162	7,850,754
11/13/17	Cement	not applicable	18:09:00	081 + 100	161,494	7,899,147
11/22/17	Kerozene	1223	23:01:00	128 + 300	176,647	7,854,871

**Table 3:** Vulnerability assessments within the watersheds that surround the studied segment of BR 050 highway (*V*), considering the four scenarios. Vulnerability assessments within the 200 m buffers that surround the 14 road accidents (also termed hazard assessments; *H*), considering the same scenarios. Risk assessments (*R = H/V*, in percent ratio).

Intercepted Basins [15]			Buffers Around Road Accident Sites		
**Scenario 1—Maximize ground slope factor**					
**Category**	**Vulnerability**		**Hazard**		**Risk—*R***
	**Area—*V* (hectare)**	**%**	**Area—*H* (hectare)**	**%**	
Invulnerable	1425.79	1.12	1.33	0.79	0.71
Weakly vulnerable	79,725.12	62.46	85.90	51.05	0.82
Vulnerable	42,135.85	33.01	69.54	41.32	1.25
Strongly vulnerable	4337.79	3.40	11.50	6.83	2.01
Extremely vulnerable	17.07	0.01	0.00	0.00	0.00
Total	127,641.62	100.00	168.27	100	
**Scenario 2—Maximize drainage density factor**					
**Category**	**Vulnerability**		**Hazard**		**Risk—*R***
	**Area—*V* (hectare)**	**%**	**Area—*H* (hectare)**	**%**	
Invulnerable	1416.06	1.11	1.33	0.79	0.7
Weakly vulnerable	31,358.31	24.57	44.59	26.50	1.1
Vulnerable	89,678.65	70.26	110.85	65.88	0.9
Strongly vulnerable	5188.61	4.06	11.50	6.83	1.7
Extremely vulnerable	0.00	0.00	0.00	0.00	nd
Total	127,641.62	100	168.27	100.00	
**Scenario 3—Maximize geology factor**					
**Category**	**Vulnerability**		**Hazard**		**Risk—*R***
	**Area—*V* (hectare)**	**%**	**Area—*H* (hectare)**	**%**	
Invulnerable	1421.19	1.11	1.33	0.79	0.71
Weakly vulnerable	25,391.51	19.89	36.89	21.92	1.10
Vulnerable	60,624.47	47.50	81.22	48.26	1.02
Strongly vulnerable	40,073.87	31.40	48.75	28.97	0.92
Extremely vulnerable	130.58	0.10	0.09	0.05	0.50
Total	127,641.62	100	168.27	100	
**Scenario 4—Maximize soil class factor**					
**Category**	**Vulnerability**		**Hazard**		**Risk—*R***
	**Area—*V* (hectare)**	**%**	**Area—*H* (hectare)**	**%**	
Invulnerable	1416.06	1.11	1.33	0.79	0.71
Weakly vulnerable	22,612.48	17.72	28.58	16.98	0.96
Vulnerable	77,085.70	60.39	100.15	59.52	0.99
Strongly vulnerable	26,190.75	20.52	37.95	22.56	1.10
Extremely vulnerable	336.63	0.26	0.27	0.16	0.62
Total	127,641.62	100	168.27	100	
